# Improving hydraulic performance of the left atrial assist device using computational fluid dynamics

**DOI:** 10.1111/aor.14850

**Published:** 2024-09-05

**Authors:** Mark S. Goodin, Chihiro Miyagi, Barry D. Kuban, Christine R. Flick, Anthony R. Polakowski, Jamshid H. Karimov, Kiyotaka Fukamachi

**Affiliations:** ^1^ Simu Tech Group Inc. Hudson Ohio USA; ^2^ Department of Biomedical Engineering Lerner Research Institute, Cleveland Clinic Cleveland Ohio USA; ^3^ Shared Laboratory Resources Lerner Research Institute, Cleveland Clinic Cleveland Ohio USA; ^4^ Cleveland Clinic Lerner College of Medicine Cleveland Ohio USA

**Keywords:** device‐based therapy, diastolic dysfunction, heart failure with preserved ejection fraction, mechanical circulatory support

## Abstract

**Background:**

The left atrial assist device (LAAD) is a novel continuous‐flow pump designed to treat patients with heart failure with preserved ejection fraction, a growing type of heart failure, but with limited device‐treatment options. The LAAD is implanted in the mitral plane and pumps blood from the left atrium into the left ventricle. The purpose of this study was to refine the initial design of the LAAD, using results from computational fluid dynamics (CFD) analyses to inform changes that could improve hydraulic performance and flow patterns within the LAAD.

**Methods:**

The initial design and three variations were simulated, exploring changes to the primary impeller blades, the housing shape, and the number, size, and curvature of the diffuser vanes. Several pump rotational speeds and flow rates spanning the intended range of use were modeled.

**Results:**

Guided by the insight gained from each design iteration, the final design incorporated impeller blades with improved alignment relative to the incoming flow and wider, more curved diffuser vanes that better aligned with the approaching flow from the volute. These design adjustments reduced flow separation within the impeller and diffuser regions. In vitro testing confirmed the CFD predicted improvement in the hydraulic performance of the revised LAAD flow path design.

**Conclusions:**

The CFD results from this study provided insight into the key pump design‐related parameters that can be adjusted to improve the LAAD's hydraulic performance and internal flow patterns. This work also provided a foundation for future studies assessing the LAAD's biocompatibility under clinical conditions.

## Graphical abstract

The “left atrial sssist device (LAAD) is a novel pump designed to treat patients with heart failure with preserved ejection fraction. The flow path of the initial design and three variations were simulated using computational fluid dynamics (CFD) which provided insight into key design‐related parameters that aided in improvements to LAAD's hydraulic performance and internal flow patterns.”

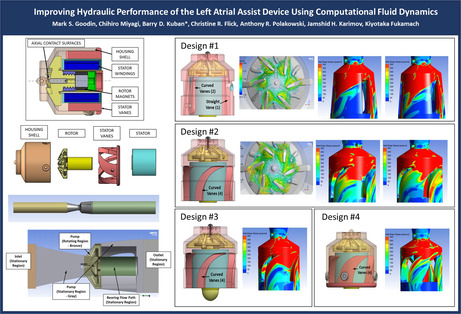

## 1 INTRODUCTION

Heart failure (HF) with preserved ejection fraction (HFpEF) accounts for approximately half of all HF.^1^ Although sodium‐glucose cotransporter‐2 inhibitors, angiotensin receptor/neprilysin inhibitors, and aldosterone antagonists showed some positive effects on HFpEF recently,^2^, ^3^, ^4^, ^5^ there are still fewer treatment options available for these patients than for those with HF with reduced ejection fraction (HFrEF). In addition, the prevalence of HFpEF has been consistently increasing, and the incidence rate ratio of HFrEF between decades (1990–1999 and 2000–2009) was 0.80 (95% CI 0.69–0.93, *p* = 0.0029) while that of HFpEF was 1.53 (95%CI 1.30–1.79, *p* < 0.0001).^6^ The prognosis and quality of life for HFpEF, however, are as poor as HFrEF.^7^, ^8^, ^9^

Many novel device‐based treatments designed specifically to treat HFpEF have been proposed over the past several years.^10^, ^11^, ^12^ The representative examples are (1) interatrial shunt devices; (2) a left ventricular (LV) expander^12^; (3) LV assist devices (LVADs) with left atrial (LA) cannulation^13^; and (4) other mechanical circulatory support devices. Among them, (1) interatrial shunt device (IASD) has progressed to the most advanced phase with receiving CE approval in Europe,^14^, ^15^ but their efficacy is limited because these devices can provide only a symptomatic treatment. For (2) and (3), only limited reports of attempts are available. Therefore, (4) device‐based therapies specifically designed to mitigate the HFpEF hemodynamics are now strongly required.

The left atrial assist device (LAAD) discussed in this report is intended to treat HFpEF under this unmet need. The LAAD is a mixed‐flow pump to be implanted in the mitral plane that pumps blood from the LA to the thickened, less compliant LV of a HFpEF heart.^16^ This pumping action is meant to reduce the left atrial pressure (LAP) and increase the LV end‐diastolic volume. The cardiac output is thus increased by the preserved ejection of the increased ventricular volume, thereby providing treatment for the diastolic dysfunction primarily observed in HFpEF populations.^17^ The initial in vitro simulation studies,^16^, ^18^, ^19^ and several in vivo studies with calves,^17^, ^20^ have shown good pump performance and functional effects in relieving high LAP caused by simulated diastolic dysfunction.

The purpose of the current study was to refine the initial LAAD design, using computational fluid dynamics (CFD) analyses and in vitro testing to inform design changes that improve the pump's hydraulic performance. Building upon the knowledge from this work, future modeling efforts will focus on predicting the hydraulic performance and biocompatibility of the LAAD under more clinically relevant conditions. Such conditions would include the effects of time‐varying changes in the patient's heart anatomy and model boundary conditions along with a non‐Newtonian viscosity model for blood.^21^, ^22^, ^23^, ^24^, ^25^

## 2 MATERIALS AND METHODS

### 2.1 LAAD designs description

**FIGURE 1:**
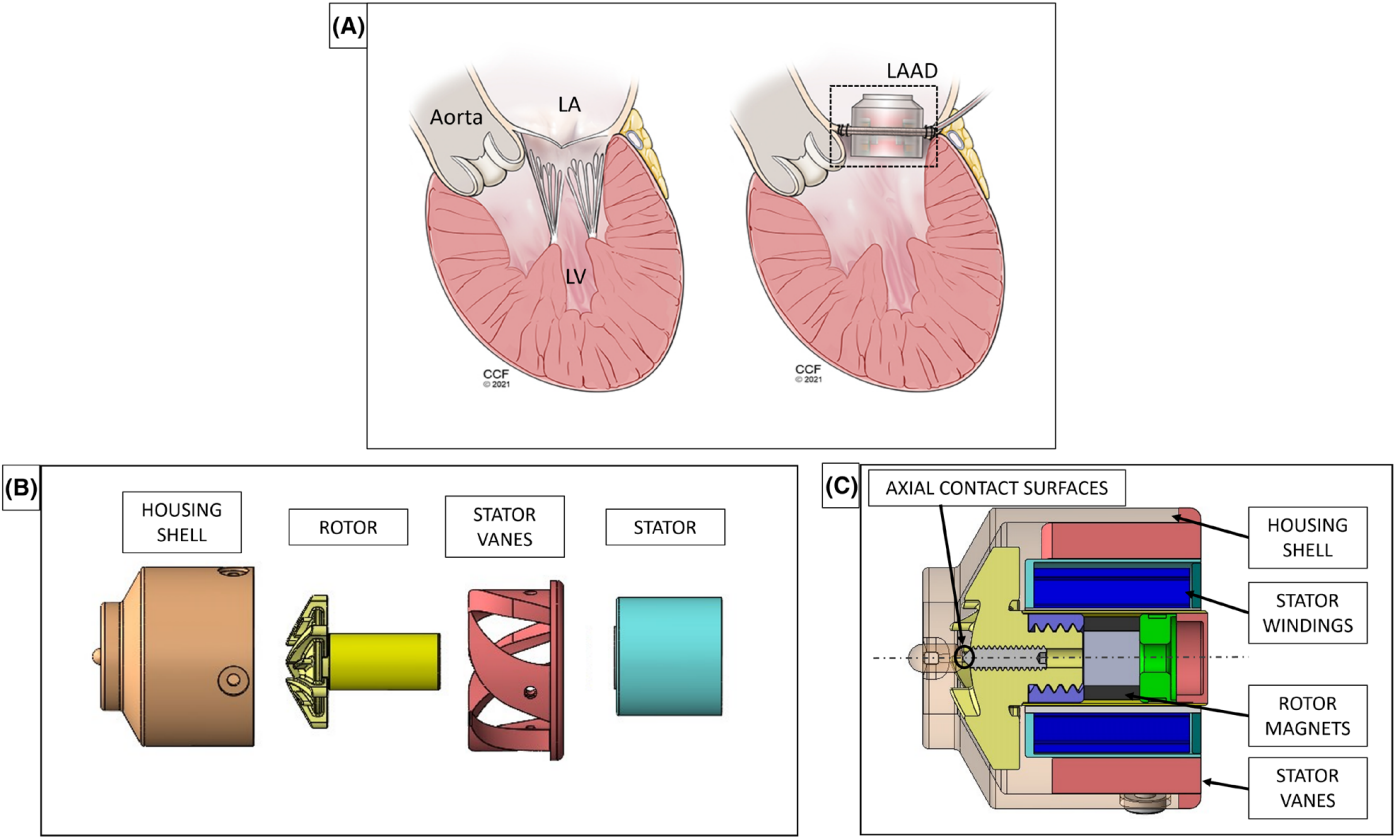
(A) A schematic illustration of the LAAD in a heart, (B) an exploded and (C) a cross‐sectional view of the initial LAAD design. [Color figure can be viewed at wileyonlinelibrary.com]

The LAAD is a continuous‐flow pump implanted at the mitral position to preserve physiological blood pathways inside the heart (Figure 1A). The LAAD functions to mitigate pathological hemodynamics of diastolic dysfunction by pumping blood from the LA to the LV, to reduce LAP and properly fill the LV.

The flow created by continuous‐flow rotary pumps (e.g., the LAAD and other continuous‐flow LVADs) can be affected by the pressure difference between the outflow and inflow (delta pressure), thus varying depending on the phase of each cardiac cycle. The main efficacy of the LAAD can be seen in diastole, as it aims to improve the LV filling and lower the LAP by creating more flow in diastole. Given this, the delta pressure in the diastole should not be very high even with HFpEF hemodynamics.^8^, ^26^, ^27^ However, because of its unique implantation at the mitral plane between the LA and LV, the LAAD must overcome higher delta pressure using higher pump speeds in the systolic phase.^20^ This is a large difference from other LVADs, which experience lower delta pressure in the systole between the LV and aorta. Therefore, although the main efficacy of the LAAD is aimed at creating more flow in the diastole, it must overcome high delta pressure in the systole as well (require 4–8 L/min at delta pressure of 60–120 mm Hg).

Exploded and cross‐sectional views of the initial LAAD design are shown in Figure 1B,C, respectively. The pump's impeller has six primary blades and six splitter blades. The primary blades extend below the impeller's conical surface to remove areas of stasis beneath the impeller and reduce the lift‐force acting on the impeller. The splitter blades assist the primary impeller blades in turning the flow and reducing flow separation between the primary impeller blades. A magnet, embedded in the rotor, is used to offset the hydrodynamic forces acting on the rotor and stabilize the rotor both axially and radially. A small contact surface is provided at the center of the impeller to limit axial movement of the rotating assembly.

**FIGURE 2:**
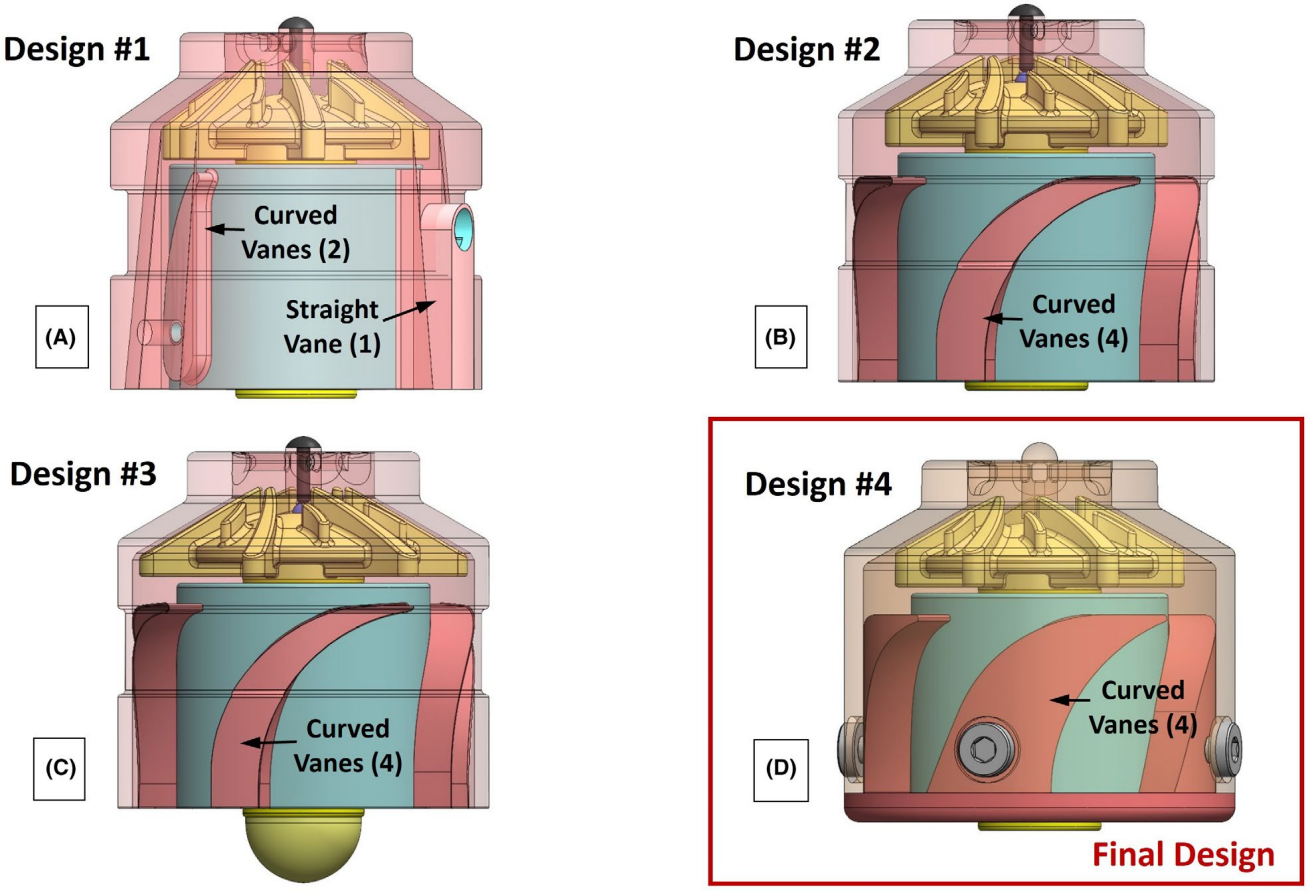
Side views of the three‐dimensional models used for the four LAAD design variations: Design #1 (A) has one straight and two curved diffuser vanes, designs #2 (B), #3 (C), and #4 (D) each have four curved diffuser vanes. Design #4 (D) is the final design. [Color figure can be viewed at wileyonlinelibrary.com]

Four different LAAD designs were evaluated in this study as shown in Figure 2. The key differences among the designs included changes to the shape and dimensions of the primary impeller blades, the housing shape, as well as to the number, size, and curvature of the diffuser vanes. A constant feature among the designs is that each impeller had six primary and six splitter blades. The key design changes for the four LAAD configurations will be presented later in the *LAAD Design Evolution* discussion within Section 3.

### 2.2 CFD modeling description

**FIGURE 3:**
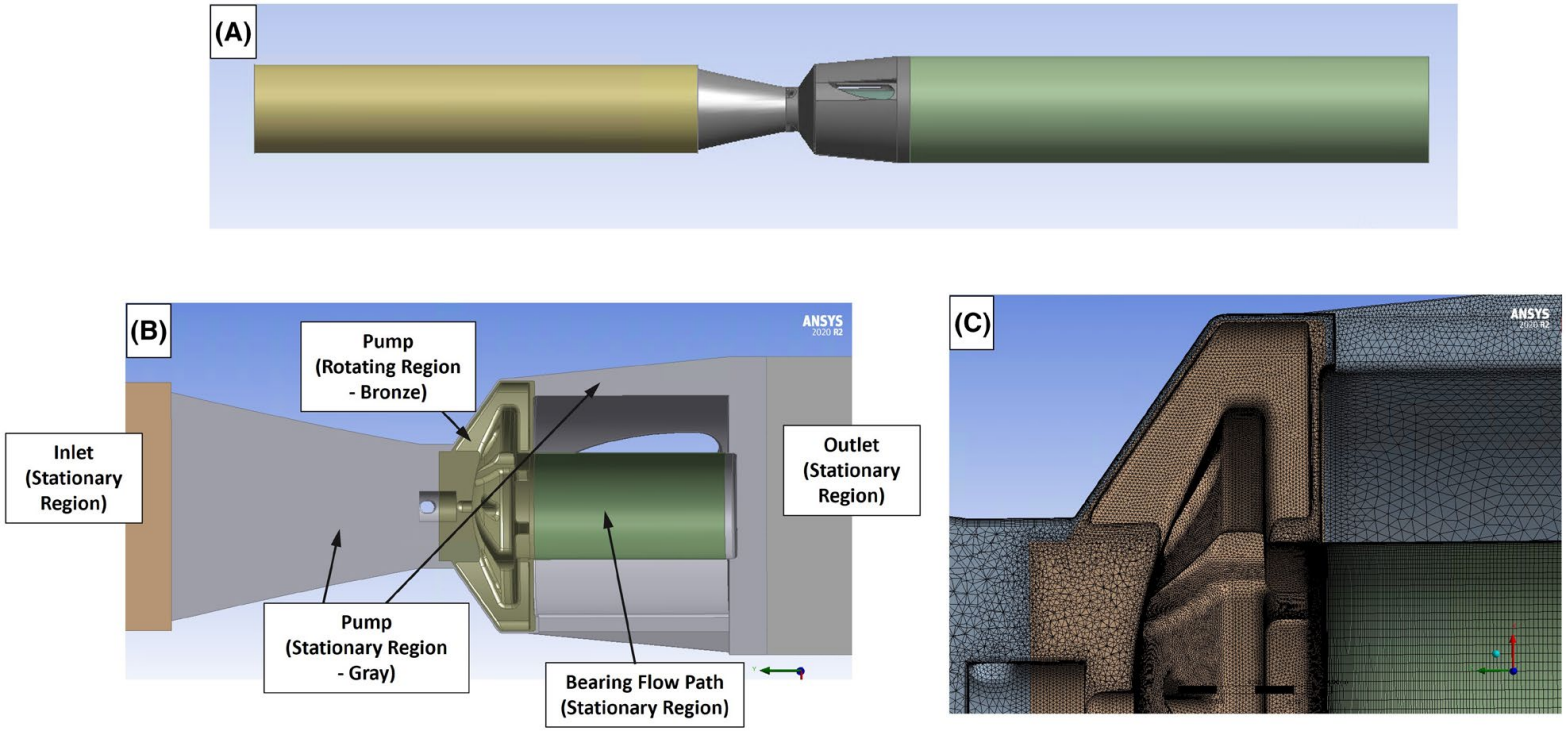
(A) An image of the CFD model for LAAD design #1, including the inlet and outlet extensions. (B) A cross‐sectional view of CFD model for design #1, showing the rotating and stationary fluid regions. (C) A close‐up view of the refined computational mesh near the primary impeller blade for design #1. [Color figure can be viewed at wileyonlinelibrary.com]

The CFD analyses were performed using Ansys CFD software (Ansys Inc., Canonsburg, PA, versions 2020R2 and 2021R2). Figure 3A illustrates the CFD model of the initial pump design. Cylindrical inlet (12.7 cm long) and outlet (15.2 cm long) extensions were added to the pump models to reduce the influence of the inlet/outlet boundary conditions and to mimic the in vitro test setup. A cross‐sectional view of the initial design CFD model, exhibiting the rotating and stationary fluid regions, is shown in Figure 3B.

A hybrid mesh, consisting of tetrahedral, prism, and hexahedral shaped elements, was used for the CFD analyses. Tetrahedral volume meshes with prism layers along the walls were used for the pump inlet, impeller, and diffuser regions. Hexahedral meshes were created for the journal bearing and the cylindrical inlet/outlet extensions. Figure 3C shows a close‐up view of the refined computational mesh near the impeller blade for design #1. The same refined mesh settings were used for all four designs. A mesh sensitivity study was performed and is discussed in Section 3.

In clinical use, the blood flow rate and LAAD rotor speed would vary constantly to meet the patients' hemodynamic needs. In addition, the LAAD outflow conditions would be constantly varying due to changes in the patient's heart anatomy. Acknowledging the time‐dependent functioning of the LAAD clinically, our current goal was to gain a solid understanding of how to improve the pump's hydraulic performance under more controlled conditions. This baseline understanding could then be applied to future work focused on both biocompatibility and the transient hydraulic performance of the LAAD. For this initial study, we therefore decided to run the CFD simulations assuming steady‐state flow conditions using the frozen‐rotor multiple frames of reference model.

In clinical use, the rotating assembly floats freely axially based on the lift forces acting on the impeller with its upward travel limited by the pivot point. For this study, we assumed the rotating assembly was centered radially and just touching the center pivot point axially. To better predict the pump's flow field under adverse pressure gradients and the onset of flow separation compared with other two‐equation Reynolds Averaged Navier Stokes turbulence models, the turbulent flow effects were captured using the shear stress transport k/omega turbulence model.^28^ The fluid material properties were set to those from the water‐glycerin mock loop data, with a density of 1060 kg/m^3^ and a viscosity of 1.90 cps. The average static pressure at the inlet was defined as 0.0 mm Hg, and the walls were considered smooth with no slip velocity. Setting an inlet pressure of 0.0 mm Hg provided a simple reference pressure from which the CFD‐predicted and in vitro test pressures could be compared. The outlet mass flow rates and the impeller rotational speeds were set to match the in vitro test conditions. Applying an inlet average static pressure and an outlet mass flow rate provided more physically realistic flow conditions at both boundaries.

A pseudo‐transient time stepping approach was used for all the simulations with a goal RMS convergence criterion for mass and momentum convergence of 0.0001. The mass RMS convergence criterion was met for all simulations with the momentum RMS convergence levels falling below 7e‐03. Impeller torque, impeller forces, impeller and housing y+ values, and static pressures at several locations were monitored. These values showed an oscillatory nature, reaching steady mean levels after approximately 100 iterations. This oscillatory nature in the monitored values is consistent with the recirculating flow regions observed in the CFD results. To help ensure that the mean values did not shift with increased iterations, we ran all the simulations out to 300 iterations.

### 2.3 In vitro studies description

The in vitro studies were conducted by connecting a prototype LAAD to a mock circulatory loop system designed to simulate various levels of diastolic heart failure (Figure S1). This mock loop included a pneumatic mock ventricle (AB5000; ABIOMED, Danvers, MA), an adjustable arterial afterload and compliance chamber, a left atrium tank, and the LAAD inserted between the left atrium and the mock ventricle. A flow probe (Transonic Systems Inc., Ithaca, NY) was used for measuring flow rate, and a Meritrans DTXPlus pressure transducer (Manufacturer Merit Medical, Singapore) was used for static pressure measurements. The testing was conducted under steady‐state flow conditions. A more detailed description of the methods used has been previously described.^17^

For the prototype devices, all components were printed in‐house. The impellers and pump housings were fabricated using a PolyJet 3D printer (J850) and made of VeroClear (Stratasys, Prairie, MN). Rounded stainless‐steel components were added at the center of the impeller and pump housing to create an axial contact surface. Small diameter‐holes were drilled into the pump housing walls to attach the static pressure taps.

## 3 RESULTS

### 3.1 Mesh sensitivity study

A mesh sensitivity study was performed on design #4 to evaluate any changes in its hydraulic performance with varying mesh refinement. Design #4 was selected, as it had the most test data for comparison and was the final, preferred design. Three different meshes were generated: coarse (8.8M elements), mid‐level (14.6M elements), and refined (24.9M elements). Eight inflation layers, with an initial layer thickness varying from 0.008 to 0.004 mm at the blade tips, were defined for the refined mesh impeller surfaces. The highest impeller speed of 4400 rpm and volumetric flow rates of 0.1, 4.5, and 7.8 L/min were simulated. The maximum pressure difference among the solutions was 4.1% occurring for the volute pressure at 0.1 L/min. The impeller torques for the coarser mesh were within 4.4% of the refined mesh values. The average and maximum y+ values decreased from 1.6 and 5.3 for the coarser mesh to 1.1 and 2.8 for the refined mesh, at the highest 7.8 L/min flow rate condition. The y+ values are a dimensionless distance from the wall based upon the local wall shear stress. Lower values of y+ indicate better resolution of the velocity gradient, and hence shear stress, at the wall.^29^, ^30^ The CFD results support that the LAAD's hydraulic performance was quite consistent among all the mesh refinement levels evaluated. For consistency, and to be conservative, the refined mesh sizing parameters were used for all the CFD models when comparing the performance of the four different LAAD designs. The number of elements in these models was 16.7M for design #1, 21.7M for design #2, and 24.7M for design #3. Design #1 had fewer elements due to its simpler diffuser design. The good agreement between CFD‐predicted static pressures and in vitro data for the tested designs #1 and #4, discussed further in Section 3, helped support the level of the mesh refinement selected for this study.

### 3.2 LAAD design evolution

Subsequent sections will describe each design, and the evolution of the LAAD flow path guided by insight gained through the CFD modeling results and in vitro test data. A summary of key component dimensional changes among the four LAAD designs is provided in Table 1.

**TABLE 1:** A summary of the key component dimensional changes among the four LAAD designs.

Description of change to model	Design #1	Design #2	Design #3	Design #4	Units
Impeller					
Primary blade height at inlet	4.7	3.7	3.7	3.7	mm
Primary blade outer diameter	25.4	25.4	29.8	25.4	mm
Primary blade inlet angle	35	20	20	20	degrees
Splitter blade inlet angle	48	40	40	40	degrees
Housing					
Number of vane blades	3	4	4	4	each
Diffuser vane blade width at outlet	4.6	3.9	3.9	13.2	mm
Diffuser vane blade inlet angle	40	75	75	75	degrees
Tapered or cylindrical diffuser section	Tapered	Cylindrical	Cylindrical	Cylindrical	–

### 3.3 First‐generation LAAD pump design

The initial LAAD design had a primary impeller blade height of 4.7 mm and an impeller outer diameter of 25.4 mm. This design also included one rectangular diffuser vane, used for the pump electrical assembly, and two curved diffuser vanes.

**FIGURE 4:**
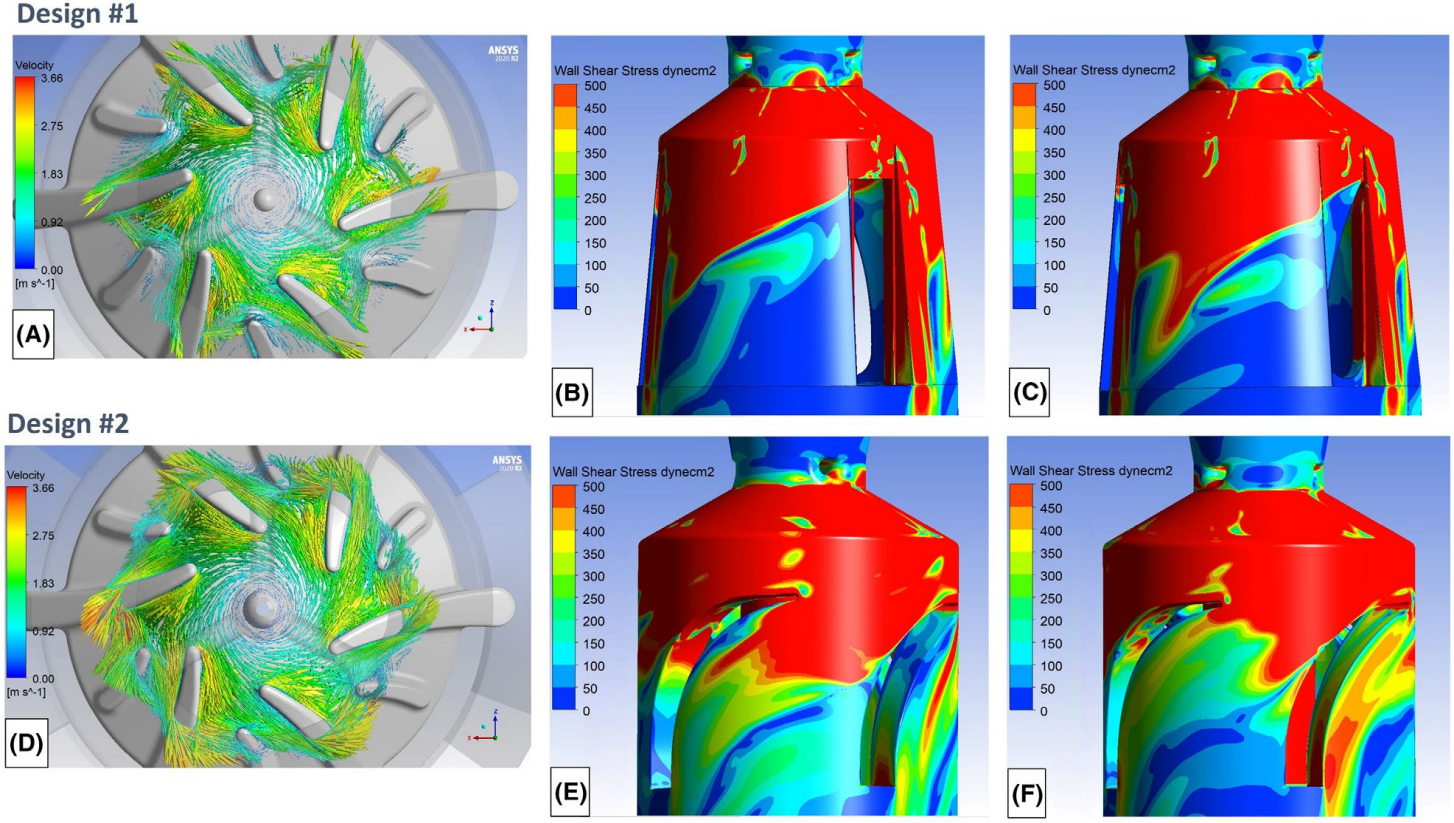
(A) A plot of velocity vectors in the rotating frame of reference on a plane mid‐blade height for design #1, revealing blade tip‐induced flow separation. (B) An image showing regions of low wall shear stress downstream of the rectangular diffuser vane for design #1. (C) An image showing regions of low wall shear stress downstream of the curved diffuser vane for design #1. (D) A plot of velocity vectors in the rotating frame of reference on a plane mid‐blade height for design #2, showing significantly reduced blade tip‐induced flow separation compared with design #1. (E) An image showing reduced regions of low wall shear stress downstream of the curved diffuser vanes for design #2 compared with design #1. (F) An image showing similar wall shear stress patterns on the opposite side of design #2 as that presented in Figure 6 (E). [Color figure can be viewed at wileyonlinelibrary.com]

The CFD results for this design revealed significant flow separation on the low‐pressure side of the primary and splitter impeller blades, downstream of their leading edges. Figure 4A, which plots velocity vectors in the rotating frame of reference on a plane mid‐blade height, illustrates this tip‐induced flow separation. Large, separated flow regions were also observed downstream of the diffuser vanes, as revealed by the low wall shear stress regions in Figure 4B (rectangular diffuser vane) and Figure 4C (curved diffuser vane). An upper value of 500 dyne/cm^2^ was selected to highlight the low wall shear stress regions.

### 3.4 Second‐generation LAAD pump design

Numerous changes were implemented in design #2 to reduce flow separation and improve the pressure recovery within the impeller and diffuser sections. These changes included: (1) reducing the height of primary impeller blades, (2) adjusting the leading‐edge angle of primary and splitter impeller blades, (3) changing from a tapered to a cylindrical diffuser, and (4) replacing the one rectangular and two curved diffuser vanes with four identical curved diffuser vanes.

First, to help reduce the fluid volume and flow recirculation between the primary impeller blades, we reduced the height of the primary impeller blades, from 4.7 to 3.7 mm. The leading‐edge angles for the primary and splitter blades were also adjusted to better align the incoming flow with the blades. Supplemental Figure 2A shows an overlay of the adjusted design #2 blade curvatures on the velocity vectors from design #1. These changes improved alignment of the impeller blades with the incoming flow, and significantly reduced the flow separation within the impeller blade passages (Figure 4D).

A similar approach was used to modify the diffuser vanes. Supplemental Figure 2B provides an overlay of the increased curvature diffuser vanes for design #2 guided by the volute streamlines from design #1. The streamlines adjacent to the impeller blades are shown in the rotating frame of reference whereas the streamlines in the stationary sections are in the absolute frame of reference. The leading edge for the diffuser vanes in design #2 were increased from 40° to 75° to better align with the primarily circumferential flow in the volute section. We also replaced the one rectangular and two curved diffuser vanes, with the four curved vanes, which improved the alignment of the diffuser vanes with the approaching volute flow. These changes reduced the leading‐edge induced flow separation and provided more uniform wall shear stresses within the diffuser as shown on opposite sides of the pump in Figure 4E,F.

Lastly, the housing shape was changed from a tapered to a cylindrical design. The intent of the cylindrical design was to expand the flow area in the volute section. The expanded flow area would help to slow down the volute flow approaching the diffuser vanes. The goal being to reduce the flow separation occurring off the leading edge of the diffuser vanes.

Incorporating these changes, the pressure recovery in the diffuser section of the pump was significantly improved with the second design, which in turn improved the pump's overall hydraulic performance. The pump pressure rise for design #2 increased by 5% at 0.1 L/min, 20% at 4.4 L/min, and 29% at 7.7 L/min at a rotor speed of 4400 rpm, relative to design #1.

### 3.5 Third‐generation LAAD pump design

**FIGURE 5:**
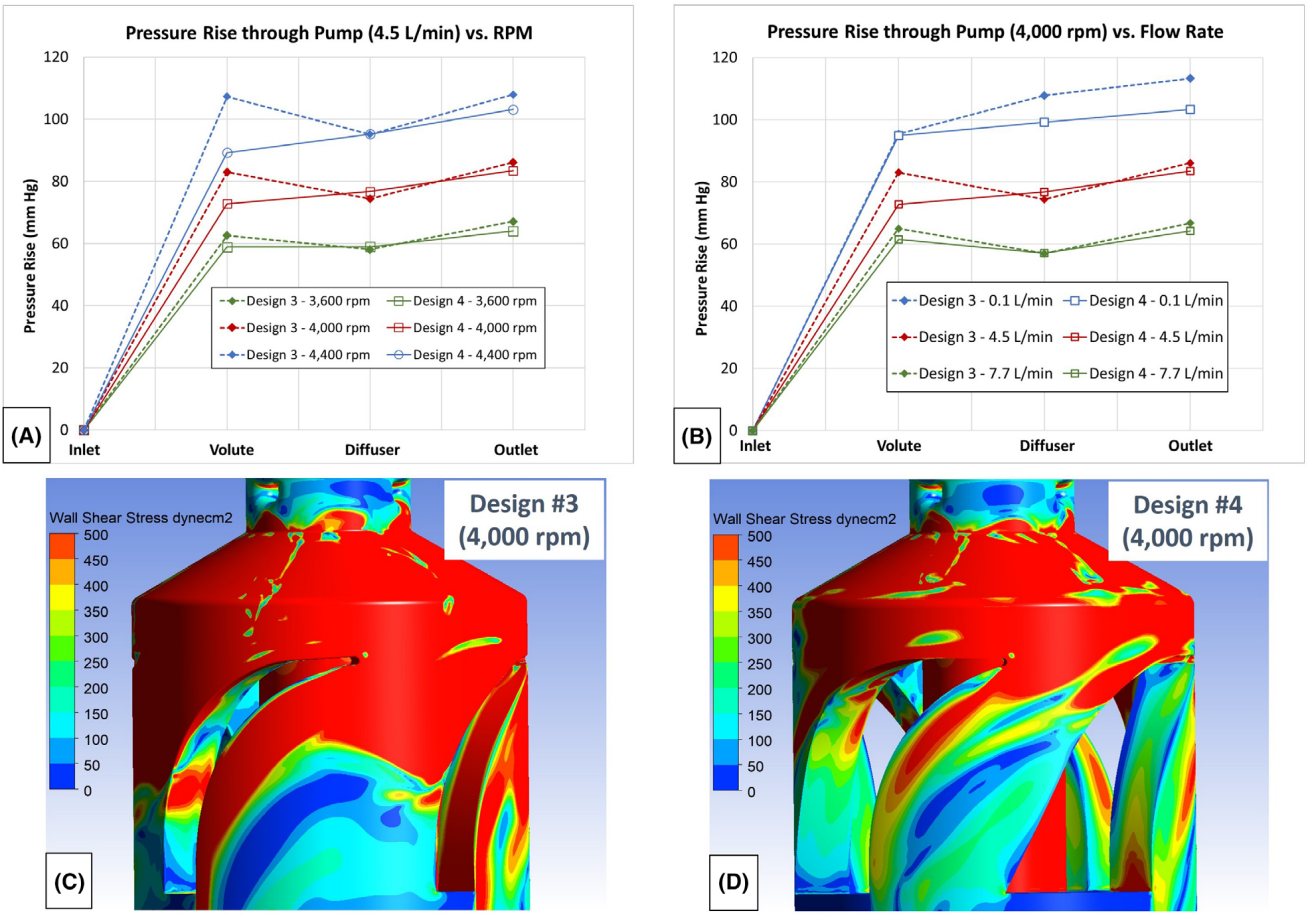
(A) Static pressure rises through the pump versus rotor speed for design #3 at 4.5 L/min flow rate. (B) Comparison of the static pressure rises through the pump versus rotor speed for designs #3 and #4 at 4.5 L/min flow rate. (C) An image showing regions of low wall shear stress downstream of the curved diffuser vanes for design #3 at a rotor speed of 4000 rpm and 4.5 L/min flow rate. (D) An image showing reduced regions of low wall shear stress downstream of the curved diffuser vanes for design #4 compared with design #3 at a rotor speed of 4000 rpm and 4.5 L/min flow rate. [Color figure can be viewed at wileyonlinelibrary.com]

For the third design, the outer diameter of the primary impeller blades was adjusted from 25.4 to 29.8 mm to take advantage of the enlarged radial space in the cylindrical housing. Changing from the tapered housing in design #1 to the cylindrical housing for designs #2 and #3 increased the volute diameter from 26.1 to 30.5 mm. The increased blade diameter enabled design #3 to achieve the same hydraulic performance (74 mm Hg at 4.5 L/min) as design 1, but at a lower rotor speed of 3750 versus 4400 rpm. Typically reducing the rotor speed is desirable as that translates into lower wall shear exerted on the blood and reduced levels of shear‐related blood damage. However, the tip speed and wall shear stress for design #3, with its larger diameter, was comparable to that for design #1. This negated the benefit of the lower rotor speed. In addition, the rotor torque required to achieve this hydraulic performance, increased significantly (63%) from design #1 (0.0075 N∙m) to design #3 (0.0122 N∙m). Lastly, the reduction in the static pressures from the volute to the diffuser section indicated that significant diffuser total pressure losses were occurring for this design at the 4.5 L/min flow rate (Figure 5A).

### 3.6 Fourth‐generation LAAD pump design

In the final design, the outer diameter for the primary blades was returned to 25.4 mm, and the width of the diffuser vanes was markedly increased, from 4.6 to 13.2 mm. The intent for the impeller changes was to reduce the impeller shear stress levels, the impeller torque (power requirements), and the flow velocities in the volute approaching the diffuser vanes. The rationale for significantly widening of the diffuser vanes near the pump outlet was to: (1) support turning the flow from a primarily circumferential direction to an axial direction, (2) provide a more constant flow area through the diffuser section, and (3) reduce flow separation and improve the total pressure recovery through the diffuser section. Widening the diffuser blades significantly reduced the total pressure losses within the diffuser, enabling design #4 to achieve very similar hydraulic performance when compared with design #3 (Figure 5B). The wider diffuser blades also helped to reduce the extent of low wall shear regions within the diffuser section as compared with design #3 (Figure 5C,D). In addition, with its smaller primary blade diameter, the rotor torque for design #4 was reduced by 38% compared with design #3, from 0.0135 to 0.0084 N∙m, at the 4.5 L/min and 4000 rpm condition.

### 3.7 CFD model validation

**FIGURE 6:**
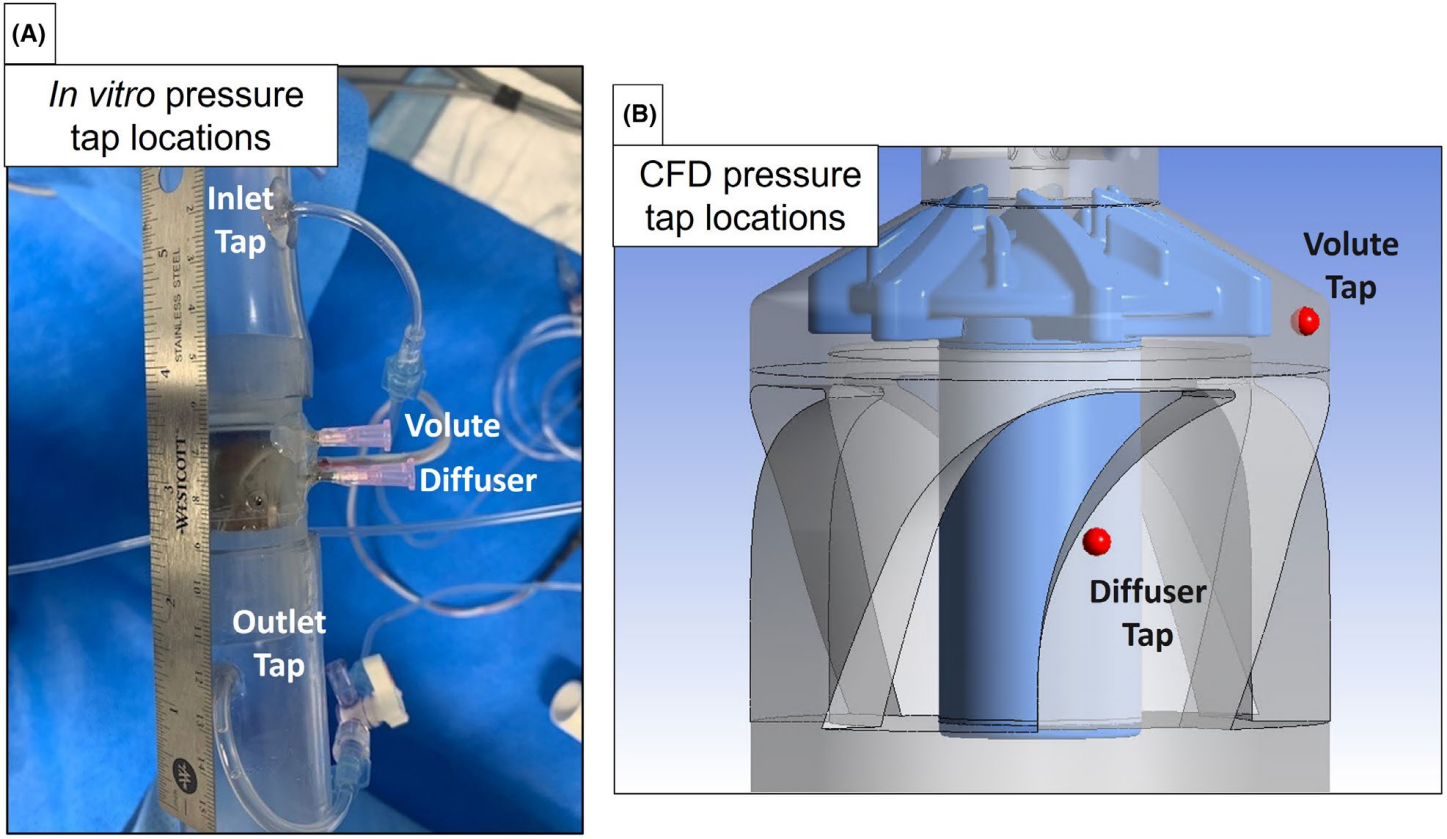
Locations for pressure tap in the in vitro experiments (A) and CFD model (B). [Color figure can be viewed at wileyonlinelibrary.com]

**FIGURE 7:**
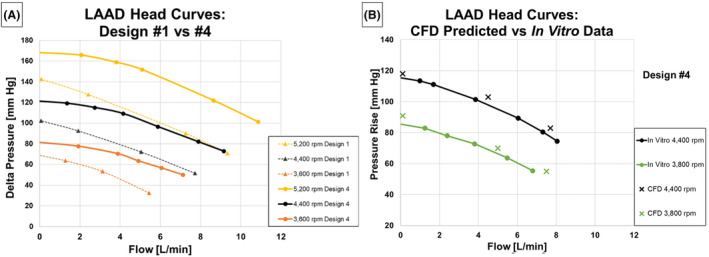
LAAD Head Curves. (A) In vitro comparison between designs #1 and #4, (B) Comparison between CFD predicted values and the in vitro study data. [Color figure can be viewed at wileyonlinelibrary.com]

The CFD‐predicted static pressures were compared to experimental values for LAAD designs #1 and #4. Given the good agreement between the CFD modeling results and the test data for design #1, and to accelerate the design iteration process, the second and third LAAD Designs were only evaluated computationally. Static pressure taps for designs #1 and #4 were placed at four locations including the inlet, within the volute just downstream of the primary impeller blades, midway down the diffuser section, and at the outlet (Figure 6). Designs #1 and #4 were tested over a range of flow rates and rotor speeds extending beyond expected clinical conditions. The head‐flow curves comparing the hydraulic performance of designs #1 and #4 are provided in Figure 7. For design #1, the CFD‐predicted pressures were compared with the in vitro measured pressures for an impeller rotational speed of 4400 rpm at a minimum flow of 0.1 L/min representing peak systole, a mean flow rate of 4.5 L/min, and a maximum flow rate of 7.7 L/min representing diastole. For design #4, the CFD‐predicted and measured pressures were compared at 3800 rpm with flow rates of 0.1, 5.0, and 7.5 L/min and at 4400 rpm with flow rates of 0.1, 4.5, and 7.7 L/min (Figure 7B).

**FIGURE 8:**
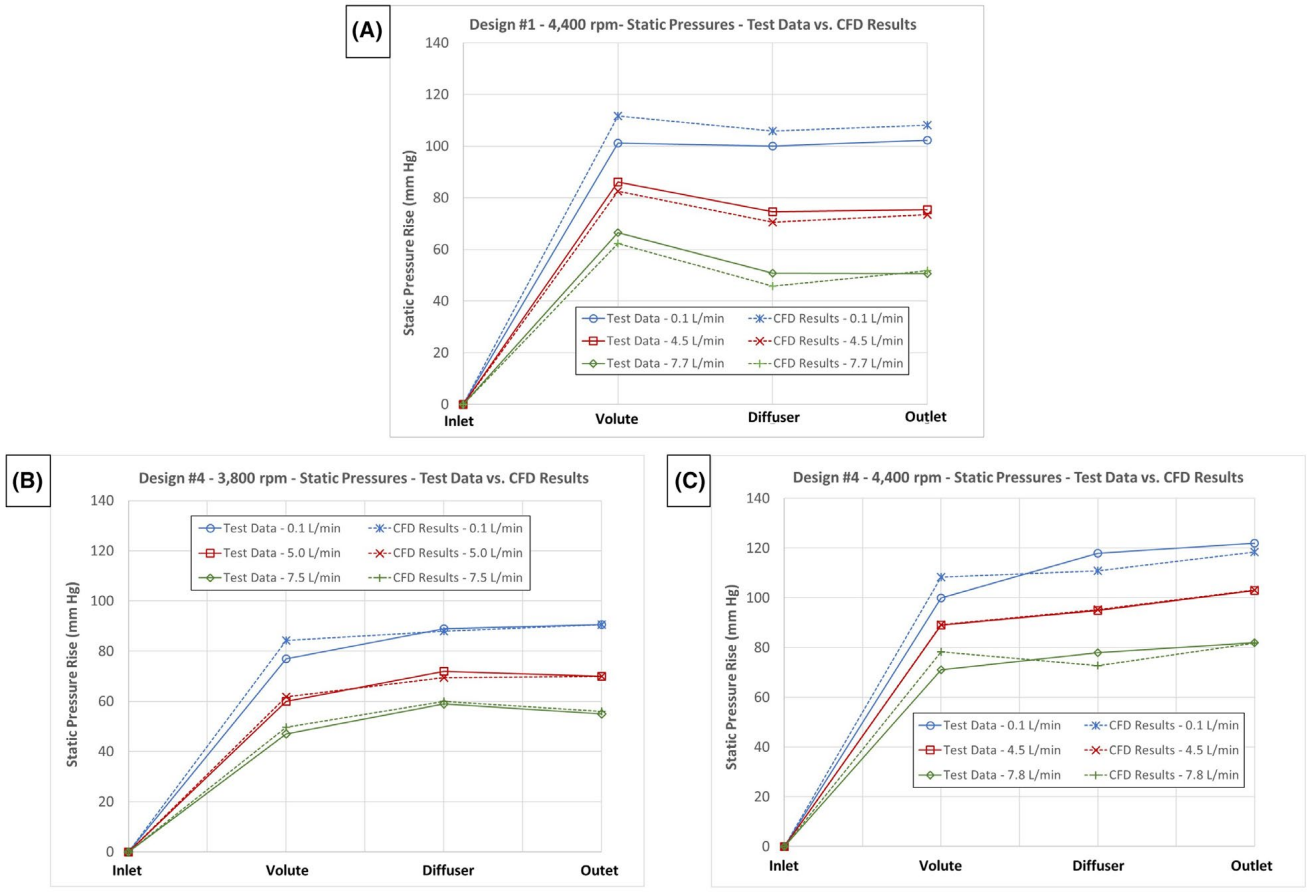
(A) Comparisons of the test data with CFD‐predicted rise in static pressures throughout the pump versus flow rate for design #1 at a rotor speed of 4400 rpm. (B) Comparisons of the test data with CFD‐predicted rise in static pressures throughout the pump versus flow rate for design #4 at a rotor speed of 3800 rpm. (C) Comparisons of the test data with CFD‐predicted rise in static pressures throughout the pump versus flow rate for design #4 at a rotor speed of 4400 rpm. [Color figure can be viewed at wileyonlinelibrary.com]

Comparisons of the static pressures throughout the pump, relative to the inlet pressure measurement for both designs, are shown in Figure 8. The volute and diffuser static pressures were averaged at four locations around the perimeter of the pump, at the axial location for each pressure tap, to reduce impact of the impeller primary blade position relative to the pressure tap. Some unsteadiness in these two static pressures was observed in the CFD results. To address this unsteadiness, the volute and diffuser static pressure values were averaged over the last 150 iterations.

For design #1, a maximum difference of 9.2% and 4.3% between the test and CFD‐predicted static pressures occurred at the volute section for the 0.1 L/min and 4.5 L/min conditions, respectively. At the pump outlet, the pressure differences were reduced to 5.5% at 0.1 L/min and 1.5% at 4.5 L/min (Figure 8A). At 7.7 L/min, the maximum pressure difference of 12.4% occurred within the diffuser section with a 3% difference in the pump outlet pressures.

With design #4, the maximum differences between the test and CFD‐predicted static pressures occurred in the volute section, being 10% at both 3800 rpm and 4400 rpm (Figure 8B,C). A higher level of agreement, within 2% at 3800 rpm and 3% at 4400 rpm, was found when comparing the outlet pressures for design #4. This level of agreement between the CFD‐predicted and measured pressures provided confidence in the modeling results when comparing the various LAAD designs.

### 3.8 Overall comparison of the LAAD design variations

**FIGURE 9:**
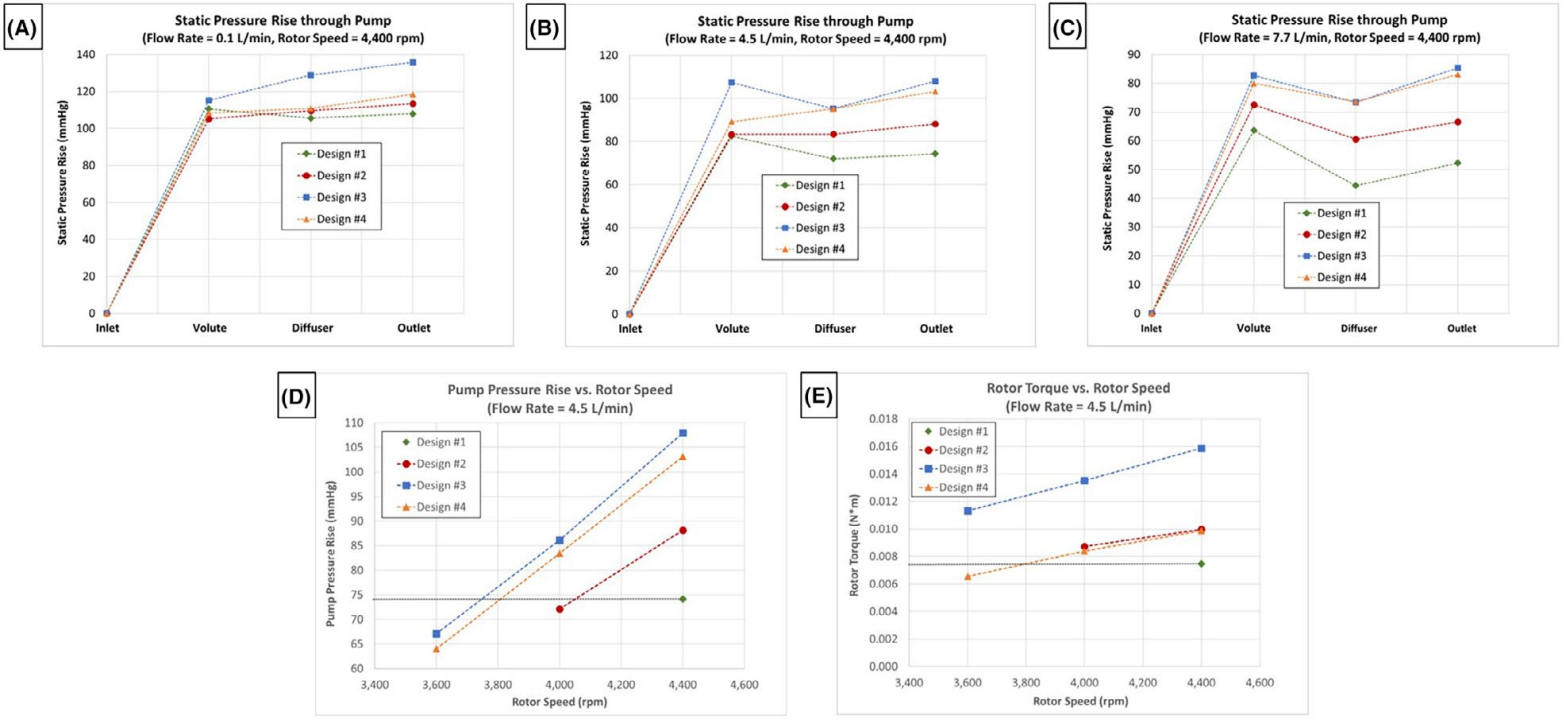
A comparison of the hydraulic performance for the four designs at a rotor speed of 4400 rpm and varying flow rates: (A) 0.1 L/min, (B) 4.5 L/min, and (C) 7.7 L/min. (D) A comparison of the overall pump pressure rises versus rotor speed for the four different LAAD design variations at a flow rate of 4.5 L/min. (E) A comparison of the rotor torques versus rotor speed for the four different LAAD design variations at a flow rate of 4.5 L/min. [Color figure can be viewed at wileyonlinelibrary.com]

A comparison of the hydraulic performance for the four designs is presented in Figure 9A–C at the 4400 rpm rotor speed condition. Design #3, with its increased impeller diameter, provides the largest pressure rise at a flow rate of 0.1 L/min (Figure 9A). Of the designs (#1, #2, #4), with the smaller 25.4 mm primary impeller outer diameter, design #4 provided the best pressure recovery within the volute section and significantly higher outlet pressures at the 4.5 and 7.7 L/min conditions (Figure 9B,C). Only in design #4 did the static pressure continue to increase through the diffuser section at the nominal 4.5 L/min flow rate (Figure 9B).

Improved pressure recovery for design #4 means that it can achieve the same pressure rise seen in design #1, but at a much lower rotor speed. At 4.5 L/min, design #4 provided a pressure rise of 74 mm Hg, with a rotor speed of approximately 3800 rpm compared with 4400 rpm for design #1 (Figure 9D). Reducing the rotor speed is important as it reduces the impeller peak shear stress levels and resultingly reduces the likelihood of blood damage. Lastly, the rotor torque for design #4 at 3800 rpm is the same as that for design #1 (Figure 9E). Providing the same torque at a lower rotor speed indicates that less power is needed to operate design #4, compared with design #1.

## 4 DISCUSSION

The focus for this study was to evaluate an initial LAAD design and explore design variations that could improve both the pump's hydraulic performance and its internal flow patterns. More specifically, we were interested in design changes that could enable the LAAD to operate at lower rotational speeds, while still achieving the desired pressure rises through the pump. Operating the LAAD at lower impeller rotational speed reduces the shear stress levels exerted on the blood which reduces the likelihood of shear‐related platelet activation and hemolysis.^21^, ^22^, ^23^, ^24^, ^25^

The initial version of the LAAD was primarily designed for ease of fabrication and to provide sufficient hydraulic performance capable of demonstrating the feasibility of the pump. Three subsequent design revisions, aimed at improving the pump's hydraulic performance and internal flow patterns for hemocompatibility, were evaluated. The CFD modeling results and in vitro test data were used to guide the evolutions of the LAAD's design modifications.

To help support the credibility of the CFD modeling, a mesh sensitivity study was conducted for design #4, which showed minor changes in the predicted hydraulic performance (<4.1%) and impeller torque (<4.4%) over the three evaluated mesh sizes. To provide the highest level of fidelity, the most refined mesh parameters were used to create the meshes for all the CFD models used in the design comparisons.

Static pressure data, obtained from an in vitro flow loop, were used to validate the CFD modeling results for designs #1 and #4. Given the good agreement between the CFD modeling results and our test data for design #1, and to accelerate our design iteration process, the second and third LAAD Designs were only evaluated computationally. The validation data included three flow rates (0.1, 4.5, and 7.7 L/min) spanning the LAAD's intended operational range of use. The CFD‐predicted and test data were compared at 4400 rpm for design #1 and both 3800 and 4400 rpm for design #4. The CFD‐predicted pressures agreed within 12% of the measured volute and diffuser pressures, and within 6% of the measured outlet pressures. This level of agreement confirmed overall confidence in the modeling methods when comparing the CFD results among the different design variations.

The three key design changes that increased the LAAD's hydraulic performance were related to improving flow alignment: (1) the curvature of the primary impeller blades was adjusted to better align the blade tips with the flow entering through the inlet, (2) the curvature of the diffuser vanes was increased to better align the vane tips with the flow approaching from the pump's volute, (3) the width of the diffuser vanes was considerably increased to help transition the flow from a predominantly circumferential direction, to a more axial flow direction. These changes reduced flow separation and greatly improved the pressure recovery, within both the impeller and diffuser sections.

One limitation of this study is that steady state, instead of transient, flow conditions were tested and modeled. In the clinical context, the blood flow rate and rotor speed would require constant adjusting to meet the patient's hemodynamic needs. While understanding the device's performance in response to these time‐varying conditions will be critical as the LAAD is further developed, the steady state method selected for this foundational study aligns well with our primary objective: understanding the key pump design‐related parameters that can be adjusted to improve the LAAD's hydraulic performance. Instead of varying the flow conditions with time, we elected to evaluate the various LAAD designs at several rotor speeds and flow rates, spanning the pump's intended range of use. This approach successfully provided insight into each design's hydraulic performance and internal flow field over a wide range of conditions. Future studies, focusing on biocompatibility, will include transient simulations to identify regions of sustained low or high wall shear stress that could lead to thrombus formation, platelet activation, and/or cell lysis.^22^, ^31^, ^32^

Another limitation is that a water‐glycerin mixture instead of blood, with its non‐Newtonian viscosity, was used in this study. As this initial study was focused on improving the baseline LAAD's hydraulic performance, we used fluid properties that matched in vitro testing conditions. Including a non‐Newtonian viscosity model will be an important next step to more accurately predict low‐shear (higher viscosity) recirculating flow regions and high‐shear (lower viscosity) regions throughout the pump which may impact the LAAD's hydraulic performance. Also, predicting the location and magnitude of low‐ and high‐shear regions will help in assessing the likelihood of thrombus formation and cell lysis, respectively.^21^, ^22^, ^23^

A third limitation was that the LAAD designs were not evaluated when inserted into an anatomically representative heart model, and straight inlets and outlets were connected to the pumps for both the testing and CFD modeling. Although it was critical to first focus on understanding and improving the LAAD's hydraulic performance, our anticipated next step will be evaluating design #4, the preferred iteration, when it is placed into a virtual mitral plane, based on a three‐dimensional scan of a patient's heart. This would allow us to better understand the impact of the heart anatomy on the LAAD's hydraulic performance and biocompatibility.

## 5 CONCLUSIONS

This study was intended to evaluate an initial LAAD design and recommend flow path design changes that could improve the pump's hydraulic performance and internal flow path. The three key design changes, identified through the CFD analyses that increased the LAAD's hydraulic performance, were related to improving the alignment of the impeller blades and diffuser vanes with the approaching flow patterns. These changes reduced flow separation and improved the pressure recovery within the pump and thus improved the overall hydraulic performance of the LAAD. Future efforts will extend to evaluate the hemocompatibility of the LAAD and tailor its flow path for use as an implanted device.

## AUTHOR CONTRIBUTIONS

All authors contributed to the design and data collection of the study. MSG conducted the CFD modeling studies and drafted the manuscript as a first author. BDK, CRF, and ARP assembled the prototype pump, and BDK, CRF, and CM performed the in vitro hydraulic performance testing. BK is the Principal Investigator of the study. KF, BDK, and JHK are co‐inventors of the LAAD. All authors critically reviewed and approved the manuscript for submission.

## FUNDING INFORMATION

This study was supported by funding from Ohio Third Frontier (The State of Ohio, Department of Development), Grant/Award Number: TVSF19‐0436.

## CONFLICT OF INTEREST STATEMENT

Kiyotaka Fukamachi, Randall C. Starling, Jamshid H. Karimov, and Barry D. Kuban are co‐inventors of the LAAD. The other coauthors have nothing to disclose.

## Supporting information


Data S1.
