# Supplementary material for: Improving hydraulic performance of the left atrial assist device using computational fluid dynamics
Source: Artif Organs. 2024 Sep 5;49(1):52–64. doi: 10.1111/aor.14850 (PMC11687210; doi:10.1111/aor.14850)
Supplement: Supplementary file 1 — Data S1. [file AOR-49-52-s001.docx]

Supplemental Materials

**
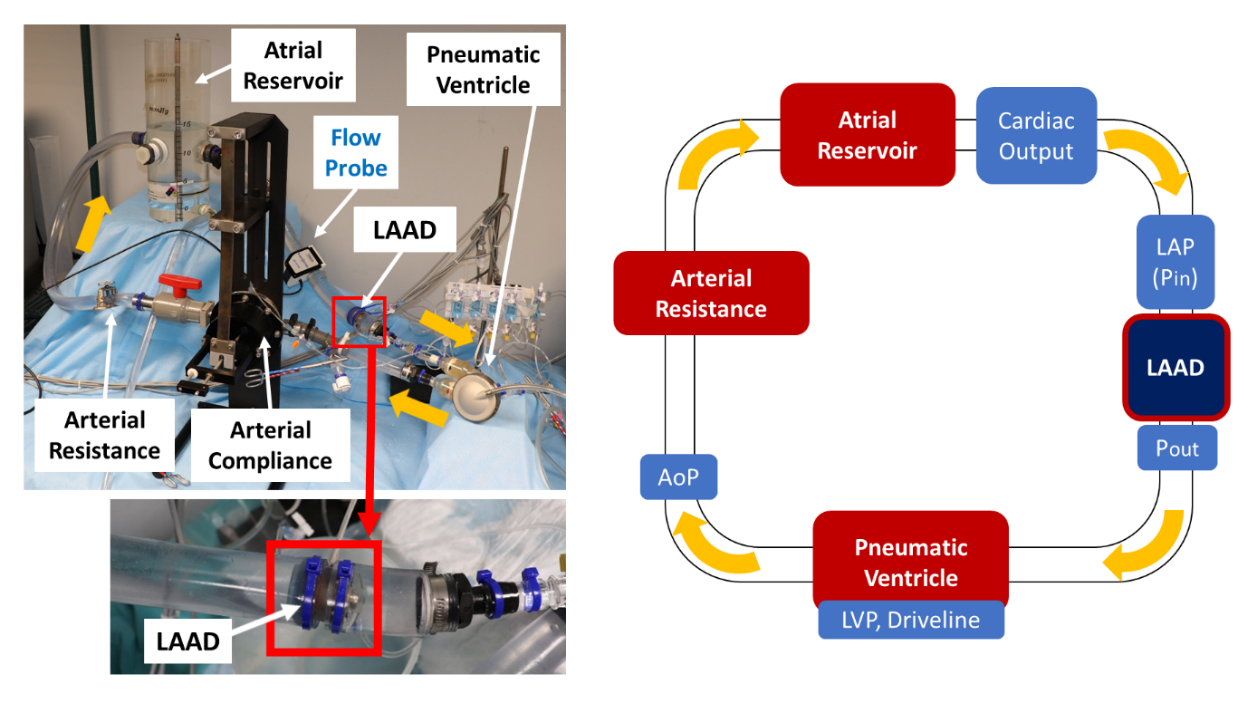
Supplemental Figure 1**

A photo (left) and schematic illustration (right) of the in vitro bench testing of the LAAD.

**
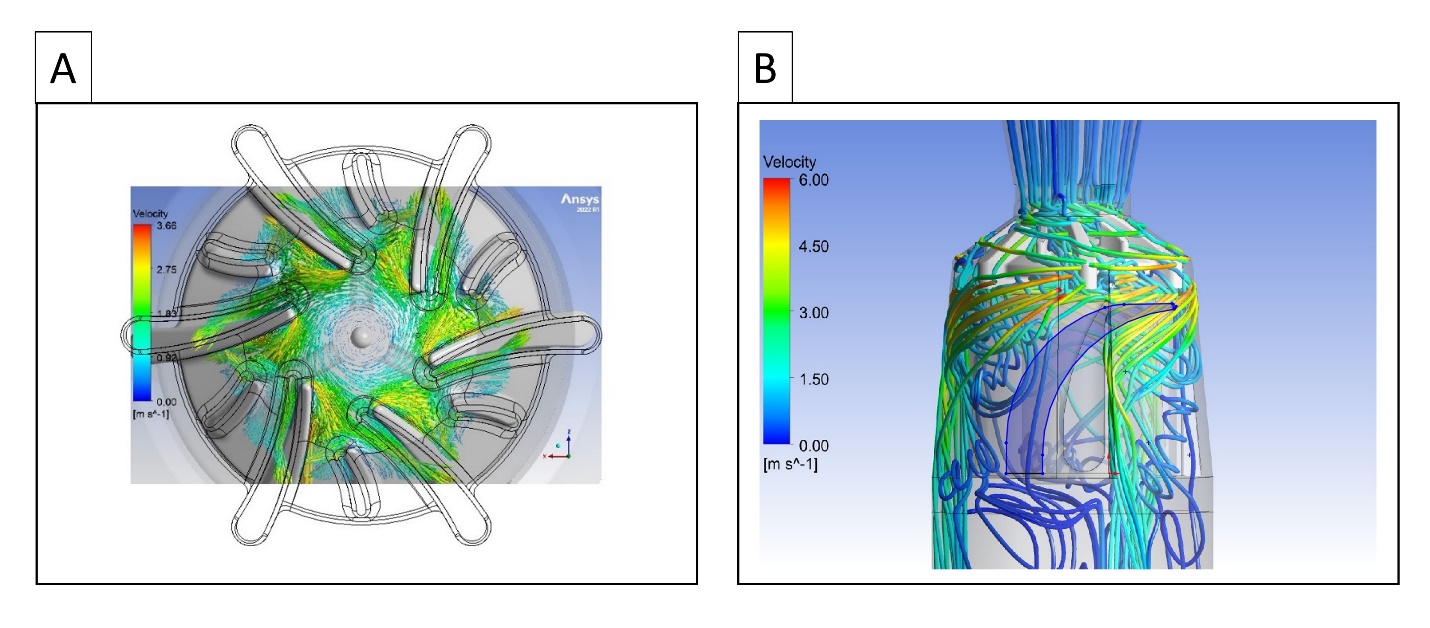
Supplemental Figure 2**

(A) An overlay of the adjusted Design #2 blade curvatures on the velocity vectors from Design #1, (B) An overlay of the increased curvature diffuser vanes for Design #2 (highlighted in blue) guided by the volute streamlines from Design #1
